# Topline results from Phase 1/2 AAV gene therapy (LX1001) in APOE4/4 homozygotes with Alzheimer’s disease

**DOI:** 10.1002/alz70859_101538

**Published:** 2025-12-25

**Authors:** Kim G Johnson, Michael Kaplitt, Stephen Kaminsky, Dolan Sondhi, Gianni Amato, Nithya Selvan, Richie Khanna, Rosalind McLaine, Sandi See Tai, Ronald Crystal

**Affiliations:** ^1^ Duke University School of Medicine, Durham, NC USA; ^2^ Weill Cornell Medical College, New York, NY USA; ^3^ Lexeo Therapeutics, New York, NY USA

## Abstract

**Background:**

APOE4 homozygotes with Alzheimer’s have faster rates of cognitive decline and become symptomatic approximately a decade earlier. APOE2 is a protective variant with reduced likelihood of developing AD and a slower rate of decline. LX1001 is an adeno‐associated viral vector investigational gene therapy (AAVrh.10hAPOE2) delivering the APOE2 gene into the central nervous system of APOE4 homozygotes to convert the brain APOE4 homozygous genotype to an APOE2/E4.

**Method:**

This is a Phase 1/2, dose escalation study (NCT03634007) evaluating the safety and tolerability of LX1001 in four ascending single‐dose cohorts (C1‐C4). LX1001 was administered into the cerebrospinal fluid (CSF) at the craniocervical junction. Enrollment criteria include APOE4 homozygotes, age ≥ 50, positive amyloid PET, CSF biomarkers consistent with AD, and mild cognitive impairment (MCI) to moderate dementia. Following the one‐time dose, the CSF APOE2/E4 profile, fluid and imaging biomarkers were assessed at regular intervals over 12 months.

**Result:**

Fifteen participants were dosed: 50% *MCI, 14% mild and 36% moderate dementia, at baseline. Twelve months of data are available for C1‐C3 and 6 months for C4. Treatment with LX1001 was generally safe and well‐tolerated. No events of amyloid related imaging abnormalities were observed*. Post‐treatment, APOE2 was expressed in CSF in all participants in a dose dependent manner. *Interim results showed stabilization in CSF Aβ42/40 and amyloid PET. There was a decrease in CSF t‐tau, p‐tau, and Tau PET. Full data results including 12‐month data for C4 will be presented during the meeting*.

**Conclusion:**

LX1001 is the first investigational gene therapy for APOE4 homozygotes. Data suggest LX1001 is generally safe and well tolerated with reduction in CSF tau biomarkers and Tau PET, measures that are highly correlated with cognitive decline.

